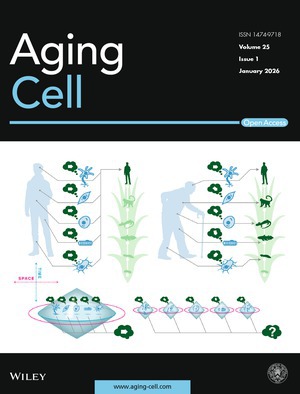# Additional Cover

**DOI:** 10.1111/acel.70420

**Published:** 2026-02-15

**Authors:** Léo Pio‐Lopez, Michael Levin

## Abstract

The cover image is based on the article *Atavistic Genetic Expression Dissociation (AGED) During Aging: Meta‐Phylostratigraphic Evidence of Cellular and Tissue‐Level Phylogenetic Dissociation* by Léo Pio‐Lopez and Michael Levin, https://doi.org/10.1111/acel.70305.